# The effect of sympathomimetic medication on cardiovascular functioning of children with attention-deficit/hyperactivity disorder

**Published:** 2009-10

**Authors:** Bianca Lee Negrao, Dalene Crafford, Margaretha Viljoen

**Affiliations:** Department of Physiology, School of Medicine, Faculty of Health Sciences, University of Pretoria; Department of Physiology, School of Medicine, Faculty of Health Sciences, University of Pretoria; Department of Physiology, School of Medicine, Faculty of Health Sciences, University of Pretoria

## Abstract

**Objective:**

The aim of this study was to investigate the effects of sympathomimetic medication on the cardiovascular system of children with attention-deficit/hyperactivity disorder (ADHD).

**Methods:**

Cardiovascular functioning of children with ADHD (*n* = 19) was tested while the children were stimulant free and during a period in which they were on stimulant medication. Electrocardiograms (ECGs) were obtained by means of a Schiller CardioLaptop AT-110 ECG recorder using the standard 12-lead cable positioning for a resting ECG. Blood pressure was measured by means of a stethoscope and mercury sphygmomanometer.

**Results:**

The main findings of this study were that methylphenidate usage is associated with increases in heart rate (HR) and blood pressure (BP), and that it does not adversely affect HR-corrected QT and JT intervals or cardiac dispersion values.

**Conclusion:**

Methylphenidate causes an increase in HR as well as increases in both systolic and diastolic BP, but no change in cardiac depolarisation and repolarisation duration or homogeneity.

## Summary

The medications commonly used to treat ADHD all have stimulatory effects on the sympathetic nervous system, and therefore they all have the potential to influence cardiovascular functioning. There is controversy about the effects of these stimulants, with recent increases in United States Food and Drug Administration (FDA) warnings causing increased concern.^1^

Stimulants commonly used to treat ADHD have been shown to cause significant increases in blood pressure compared to placebo,^2^-^4^ with methylphenidate being demonstrated by Ballard *et al.*^5^ to cause significant increases in both systolic and diastolic blood pressure. Furthermore, these stimulants have been seen to cause increases in HR,^2^-^4^ with methylphenidate being shown by Ballard *et al.*^5^ and Spencer *et al.*^6^ to cause increases in HR values. There are indications that the cardiovascular side effects of the medications commonly used to treat ADHD include an average increase of one to two beats per minute (bpm) in HR and rises of 3 to 4 mmHg in both systolic and diastolic BP.^7^ Although these side effects are believed to be insignificant for most children, they could potentially result in cardiovascular incidents in children with structural heart abnormalities associated with sudden cardiac death, such as cardiac arrhythmias, cardiomyopathies, long-QT syndrome, short-QT syndrome, Brugada syndrome, coronary artery anomalies, primary ventricular fibrillation or tachycardia, Wolff-Parkinson-White syndrome or Marfan syndrome.^7^

Studies on the effect of stimulant medication on QT and QTc intervals are contradictory, with some studies indicating a small but insignificant increase,^8^,^9^ others finding no changes,^10^,^11^ and a few suggesting a decrease in QT intervals^2^ with stimulant usage. Similarly, some studies have indicated a small but insignificant increase,^8^ others have found no changes^2^,^4^ and a few have suggested a significant increase in QTc intervals^9^ with stimulant usage. Regarding the effect of stimulant medication on QT dispersion values, some studies have indicated a decrease in QTd,^12^ while others have reported no changes in QTd values.^4^ Methylphenidate has been shown by both Ballard *et al*.^5^ and Spencer *et al*.^6^ to cause no changes in ECG parameters. No studies could be found that examined the effect of methylphenidate on JTc and JTd values.

Worryingly, indications are that ADHD may actually be more prevalent in children with heart disease.^7^ Studies have reported that 33 to 42% of paediatric cardiac patients also suffer from ADHD.^13^ Yet, in most countries, adrenergic stimulants are prescribed to children with ADHD without prior cardiovascular assessment and monitoring. The aim of this study was to investigate the effects of methylphenidate on the cardiovascular system of children with ADHD.

## Methods

Ethical clearance was obtained from the Faculty of Health Sciences Research Ethics Committee, University of Pretoria, clearance number S30/2007, and the South African Department of Health, DOH trial number DOH-27-0808-1816. Patient recruitment and supervision was conducted by a registered psychiatrist. Only children from whom and from whose guardians/parents voluntary informed consent could be obtained were included in the study.

## Subjects

The experimental group consisted of 19 children, diagnosed with ADHD by a registered psychiatrist, according to the text-revised fourth edition of the *Diagnostic and Statistical Manual of Mental Disorders* (DSM-IV-TR) (13 boys and six girls, aged six to 15 years, mean 9.53 years).

Exclusion criteria included children with co-morbidities, those on medication other than Ritalin, children with overt malnutrition, mentally retarded children and those with an inability to understand and give informed assent. Children with ADHD were regarded as being on medication if they had been taking Ritalin (methylphenidate) consistently for at least 10 days at the dosage prescribed specifically for them by their psychiatrist. Eighteen of the children with ADHD tested in this study were taking shortacting Ritalin at a dosage of 10 mg/day, while one child was on long-acting Ritalin at a dosage of 20 mg/day.

These same children were tested after they had refrained from taking Ritalin for a period of at least three weeks during their school holidays and were then considered to be stimulant free. The practise of taking children with ADHD off their stimulant medication during their school holidays is normal and was not introduced into the treatment regime as part of the study.

## Cardiovascular functioning

An average of three readings for BP, measured by means of a stethoscope and mercury sphygmomanometer, was obtained for each child. ECGs were obtained by a 12-lead Schiller CardioLaptop AT-110 ECG recorder while the children were in a supine position in a quiet environment at a constant room temperature. All children were instructed to lie quietly for five minutes before the ECGs were recorded, to allow stabilisation of ECG parameters. To minimise diurnal variations in ECG parameters, all ECGs were performed in the morning between 08:00 and 12:00.

Electrode positioning followed the standard 12-lead cable positioning for a resting ECG. The speed of the ECG trace was set at 25 mm/s while ECG trace sensitivity was set at 10 mm/mV. A general filter was set at 50 Hz to suppress AC interference without distorting the ECG. Both myogram and baseline filters were used to reduce muscle-induced noise and baseline fluctuations, respectively. A 10-second printout was obtained for each subject.

All ECG analysis was done manually. The QT interval was measured from the Q wave initiation to the terminal inscription of the T wave, i.e. the intersection of the T wave with the isoelectric line. If the Q wave was absent, the QT interval was measured from the beginning of the R wave. Extrapolation with a tangent was used if the end of the T wave was not clear. Furthermore, if the T wave was followed by a U wave, the QT interval was measured to the nadir between the T and U waves. The average of the QT intervals from three consecutive complexes from both leads II and V6 were used for the analyses.

QTc was calculated using Wernicke’s formula for HR correction in children and adolescents, namely:^14^

$QTc=\;\frac{QT}{{RR}^{0.38}}$

as well as Bazett’s formula for HR correction,^15^-^17^

$QTc=\;\frac{QT}{{RR}^{0.5}}$

In both instances, the RR interval in seconds was used. The RR interval was measured manually from the R wave of one QRS complex to the R wave of the successive QRS complex. The average RR interval from three successive complexes from both leads II and V6 was used for the analysis. QT dispersion was determined by calculating the difference between QT intervals of leads II and V6, while QTc dispersion was determined by calculating the difference between HR-corrected QT intervals of leads II and V6, with QT intervals corrected for HR using Wernicke’s formula:

$QTc=\;\frac{QT}{{RR}^{0.38}}$

The JT interval was measured from the J point, which is found at the intersection of the QRS and ST waves, to the terminal inscription of the T wave. As with the QT interval, extrapolation with a tangent was used if the end of the T wave was not clear and if the T wave was followed by a U wave, the JT interval was measured to the nadir between the T and U waves. Three consecutive JT intervals from both leads II and V6 were used for the analyses. JTc was calculated using Wernicke’s formula:

$JTc=\;\frac{JT}{{RR}^{0.38}}$

as well as Bazett’s formula:

$JTc=\;\frac{JT}{{RR}^{0.5}}$

with RR measured in seconds.

As previously stated, RR was measured manually and the average from three successive complexes from both leads II and V6 was used for the analysis. JT dispersion was determined by calculating the difference between JT intervals of leads II and V6, while JTc dispersion was determined by calculating the difference between HR-corrected JT intervals of leads II and V6, with JT intervals corrected for HR using Wernicke’s formula:

$JTc=\;\frac{JT}{{RR}^{0.38}}$

## Statistical analysis

All data were statistically analysed using Stata™ data analysis software. A paired *t*-test and the Wilcoxon signed-rank test were used to compare values obtained while the children with ADHD were on stimulant medication with those obtained while they were stimulant free.

## Results

When comparing the BP values obtained while the children with ADHD were on stimulant medication with those while they were stimulant free, statistically significant differences were found regarding systolic (*p* < 0.001) and diastolic (*p* = 0.02) BP. While they were on stimulant medication, the children demonstrated significantly higher values for both systolic (111.53 ± 7.93 mmHg vs 95.00 ± 7.64 mmHg) and diastolic (69.74 ± 8.77 mmHg vs 65.95 ± 5.97 mmHg) BP.

When comparing the ECG values obtained while the children with ADHD were on stimulant medication to those obtained while they were stimulant free, statistically significant differences were obtained for RR intervals (*p* = 0.003), HR (*p* = 0.007), QT intervals (*p* < 0.001) and JT intervals (*p* < 0.001). HR was higher in children with ADHD while they were on stimulant medication (83.95 ± 15.22 vs 74.32 ± 8.53 bpm), whereas RR (0.70 ± 0.10 vs 0.81 ± 0.11 sec), QT (358.25 ± 23.96 vs 383.95 ± 18.46 msec) and JT intervals (282.89 ± 18.25 vs 305.44 ± 13.11 msec) were longer in children with ADHD while they were stimulant free. No statistically significant differences in HR-corrected QT and JT intervals and dispersion values were found.

## Discussion

In this study, the effect of methylphenidate on the cardiovascular functioning of children with ADHD was examined by looking at systolic and diastolic BP, HR, QT, JT, QTc, JTc, QTd and JTd.

QT represents the interval from the beginning of the Q wave to the end of the T wave, and therefore corresponds to ventricular depolarisation and repolarisation. Similarly, JT represents the interval from the J point, which signifies the end of ventricular depolarisation, to the end of the T wave, and therefore corresponds to ventricular repolarisation. QT and JT intervals are, however, dependant on HR and are therefore corrected in order to compensate for individual differences in HR. Therefore, QTc and JTc are analysed in order to determine whether ventricular abnormalities are present.

QTc is commonly used as a measure of the depolarisation and repolarisation of the ventricles of the heart and is thus a surrogate marker for the risk of adverse cardiac events, and in severe cases, sudden death.^9^ A prolonged QTc interval, indicative of a prolonged cardiac repolarisation, has indeed been associated with cardiac arrhythmias^9^,^18^ and an increased risk of morbidity and mortality.^19^ However, a more generally accepted absolute index of cardiac repolarisation, which is believed to be depolarisation independent, is the HR-corrected JT interval, i.e. the JTc interval.

Formulas used to correct QT and JT intervals include linear, logarithmic, square-root and exponential equations.^19^ Correction methods such as Bazett’s formula,^15^-^17^ Fridericia’s formula^14^ and Rautaharju algorithms^20^ are commonly used. However, no universally accepted method exists. Bazett’s formula is believed to work well with heart rates between 50 and 90 bpm, but over-corrects at low heart rates and under-corrects at high heart rates.^14^ Fridericia’s formula, on the other hand, under-corrects at low heart rates and over-corrects at high hearts rates, such that the over-correction at high heart rates may lead to QTc values which are artificially low.^14^

A study by Wernicke *et al.*^14^ has, however, indicated that QT correction methods developed specifically for adults do not apply to children, since it is known that QT intervals increase with age. A meta-analysis involving 2 288 children and adolescents with ADHD^14^ found the most appropriate QT correction formula for children and adolescents to be

$QTc=\;\frac{QT}{{RR}^{0.38}}$

where RR represents the length of the entire cardiac cycle in seconds. This data-derived method is based on linear regression techniques where the optimum correction factor determined was that value which resulted in zero correlation between the QTc and RR values.^14^

Another value commonly used in the assessment of cardiac functioning is QT dispersion (QTd), which is a measure of interlead variations in QT interval lengths of the surface 12-lead ECG.^21^ QTd is therefore a non-invasive marker of underlying inhomogeneity of myocardial repolarisation.^22^ An increased QTd reflects cardiac instability and an increased risk for cardiac arrhythmias.^21^ Increased QTd values have been found in cardiac disorders such as the long-QT syndrome, drug toxicity and dilated and hypertrophic cardiomyopathies.^23^ Interestingly, Glancy *et al.*^24^ discovered that an increase in QTd values, measured four weeks after a myocardial infarction, was associated with an increased rate of mortality in the next five years. Similarly, JT dispersion is a measure of inter-lead variations in JT interval lengths of the surface 12-lead ECG and is also commonly used to detect repolarisation abnormalities and cardiac instability.^25^

The BP results from this study support the findings by Biederman *et al.*,^2^,^3^ Wernicke *et al.*,^4^ and Ballard *et al.*.,^5^ which indicated that stimulant usage is associated with increases in both systolic and diastolic blood pressure. These findings therefore confirm the sympathomimetic properties of stimulants commonly used to treat ADHD.

The ECG results from this study support previous findings by Ballard *et al.*.^5^ and Spencer *et al.*,^6^ which indicated that methylphenidate usage is associated with an increase in HR. The shortening of the QT and JT intervals is expected due to the increase in HR caused by methylphenidate usage. However, it would appear that methylphenidate does not adversely affect QTc and JTc values or dispersion values. These results therefore support the previous findings by Ballard *et al.*^5^ and Spencer *et al.*,^6^ which indicated that methylphenidate does not affect ECG parameters.

## Limitations of the study

One of the limitations of the study was that only 19 children were tested. It is also suggested that further studies should include prior testing in order to exclude children with possible co-existing learning disabilities. In this study, as in most other studies, the effect of methylphenidate on cardiovascular functioning was assessed in the supine position, at rest. To get a better picture of the influence of the drug on the cardiovascular system, the assessment should be done during the exercise stress test, or at least during an orthostatic challenge. In addition, it is suggested that the time between methylphenidate intake and ECG tracing be standardised, as it has been shown that QT dispersion decreases suddenly after methylphenidate intake, but only during the acute period shortly after its administration.^12^

## Conclusion

Methylphenidate usage is associated with an increase in HR and increases in both systolic and diastolic BP, but no change in the duration and homogeneity of cardiac depolarisation and repolarisation. The significant increases in HR and systolic as well as diastolic BP are of concern in children with cardiovascular abnormalities. This highlights the need for cardiovascular testing of children with ADHD prior to the prescription of adrenergic stimulants, as well as cardiovascular monitoring during stimulant treatment. It is further suggested that the ECG should be assessed during a physical test such as an exercise or orthostatic challenge, as it is known that cardiac defects often do not present in the supine, resting state.
